# Components of Goutengsan in Rat Plasma by Microdialysis Sampling and Its Protection on A*β*_1–42_-Induced PC12 Cells Injury

**DOI:** 10.1155/2017/7593027

**Published:** 2017-03-02

**Authors:** Hou-Cai Huang, Chun-Fei Wang, Jun-Fei Gu, Juan Chen, Xue-Feng Hou, Rong-Ling Zhong, Zhi Xia, Di Zhao, Nan Yang, Jing Wang, Xiao-Bin Tan, Xiao-Bin Jia, Liu-Qing Di, Zi-Bo Dong, Liang Feng

**Affiliations:** ^1^Affiliated Hospital of Integrated Traditional Chinese and Western Medicine, Nanjing University of Chinese Medicine, Shi Zi Street No. 100, Hongshan Road, Jiangsu, Nanjing 210028, China; ^2^Key Laboratory of New Drug Delivery System of Chinese Materia Medica, Jiangsu Provincial Academy of Chinese Medicine, Shi Zi Street No. 100, Hongshan Road, Jiangsu, Nanjing 210028, China; ^3^School of Pharmacy, Anhui University of Chinese Medicine, Hefei 230012, China; ^4^School of Pharmacy, Nanjing University of Chinese Medicine, Nanjing 210023, China; ^5^School of Traditional Chinese Medicine, China Pharmaceutical University, Nanjing 211198, China; ^6^Jumpcan Pharmaceutical Co., Ltd., Taixing 225400, China

## Abstract

Goutengsan, a Chinese herbal formula, potential protection on Alzheimer's disease (AD) has been less reported. In current study, we investigated the protection of Goutengsan on A*β*_1–42_-induced pheochromocytoma-derived cells (PC12). Furthermore, the components from Goutengsan in rat plasma were identified by microdialysis (MD) for* in vivo* sampling. Meanwhile, the protection of components identified was also verified. At last, we found that Goutengsan has a potential protective effect on A*β*_1–42_-induced PC12 cells via reducing cells damage and increasing cells vitality as well as six components (pachymic acid, liquiritin, rhynchophylline, isorhynchophylline, corynoxeine, and isocorynoxeine) which may be effective components. This study helps to understand the treatment of Goutengsan for AD and would facilitate the clinical and further studies for this formula.

## 1. Introduction

Alzheimer's disease (AD), also known as senile dementia, was a neurodegenerative disease and influenced a population of approximately 26 million worldwide with the number growing twice in 20 years [1]. The production and accumulation of amyloid *β* (A*β*) were proved to be the central pathogenesis of AD, particularly soluble A*β* oligomers which had been characterized by the progressive decline of cognitive function and behavioral derangement [2, 3]. Nowadays, the mainstream medication for AD is typically acetylcholine esterase inhibitor (AchEI), but symptomatic improvement is limited [4].

Traditional Chinese medicine (TCM) has been used for preventing and treating cognitive decline and the anti-AD agents have been made to develop from TCM for a long time [5–8]. Goutengsan, a Chinese herbal formula, consists of 11 medicinal herbs including Gouteng, Chenpi, Maidong, Banxia, Fuling, Renshen, Fangfeng, Juhua, Shengjiang, Gancao, and Shigao (Table 1). It has remarkable functions on chronic headache and hypertension [9]. Studies have suggested that Goutengsan has a function on AD and its antidementia effect was due to antihypertensive, free radical scavenging, and antiexcitotoxic effects, which were attributed to phenolic compounds and indole alkaloids [9–11]. Our early exploratory research also indicated that Goutengsan had a protective effect on AlCl_3_-induced AD rats via improving brain index [10], and Goutengsan also had obvious protective effect on glutamic-acid-induced and H_2_O_2_-induced PC12 cells injury [12]. Therefore, the protection of Goutengsan on AD treatment is worthy of further study.

Microdialysis (MD), a well-established technique for the sampling of the extracellular space, has the merits of sampling for continuous and simultaneous monitoring of changes in the local biochemical environment [13, 14]. This technique uses a catheter consisting of the perfusate, the surrounding medium, and substances, surrounded by a semipermeable membrane, which is perfused by fluid and inserted into a region of interest [15]. In recent years, MD has been proposed for the applications of pharmacological and pharmacokinetic studies. Thus, in this study, we investigated the protection of Goutengsan on A*β*_1–42_-induced pheochromocytoma-derived cells (PC12). Furthermore, the components from Goutengsan in rat plasma were identified by MD for* in vivo* sampling. Meanwhile, the protection of components identified was also verified.

## 2. Material and Methods

### 2.1. Chemicals and Reagents

Ringer's solution, containing 122 mM sodium chloride, 3 mM potassium chloride, 0.4 mM monopotassium phosphate, 1.2 mM magnesium sulfate, 25 mM sodium bicarbonate, and 1.2 mM calcium chloride, was purchased from Millipore (MA, USA). Pachymic acid, liquiritin, rhynchophylline, isorhynchophylline, corynoxeine, and isocorynoxeine (purity ≥ 99%) were purchased from National Institutes for Food and Drug Control (Beijing, China). Fetal bovine serum (FBS) was purchased from Gibco/BRL (Grand Island, NY, USA). Methyl thiazolyl tetrazolium (MTT), dimethyl sulfoxide (DMSO), and trypsin were provided by Sigma Chemical (St. Louis, MO, USA). A*β*_1–42_ (purity ≥ 95%) was purchased from Abcam (Cambridge, USA). HPLC-grade acetonitrile was purchased from Thermo Fisher Scientific (Massachusetts, USA). EliVision plus and DAB kits were provided by Fuzhou Maixin Biotech. Co., Ltd. (Fuzhou, China). Bax, Bcl-2, caspase-3 and caspase-9 antibodies, annexin V-FITC/PI assay kit, basal DMEM medium, cell lysis solution, and 0.1% DEPC water were purchased from Nanjing KeyGen Biotech Co., Ltd. (Nanjing, China). Nimodipine was provided by Bayer healthcare Co., Ltd. (Leverkusen, Germany).

### 2.2. Preparation of Goutengsan

Goutengsan consists of 11 medicinal herbs (Table 1) identified by Dr. Xiao-Bin Tan from Jiangsu Provincial Academy of Chinese Medicine (Nanjing, China) which were purchased from Anhui Huqiao Chinese Medicine Technology Co., Ltd. (Tongling, China). The remaining voucher specimens were deposited at the Jiangsu Provincial Academy of Chinese Medicine. Gouteng, Chenpi, Maidong, Banxia, and Fuling, 300 g for each; Renshen, Fangfeng, and Juhua, 200 g for each; Shengjiang and Gancao, 100 g for each; and 500 g Shigao were extracted together by refluxing with boiling water. They were boiled with 24 L water twice with 1.5 h for each time. Finally, all extract fractions were pooled and concentrated to 350 mL by rotary evaporator. The concentrated solution was stored at 4°C until use.

### 2.3. Cell Culture

Rat pheochromocytoma-derived cell line (PC12) was purchased from Institute of Biochemistry and Cell Biology (Shanghai, China). Cells were cultured in DMEM medium with 10% FBS, containing 100 units/mL of penicillin and 100 units/mL of streptomycin. Then cells were cultured in an incubator at 37°C with 5% CO_2_ and the medium should be replaced every 2 days. After 90% confluence, cells were digested by 0.25% trypsin-0.02% EDTA for the passage. A cell density of 1 × 10^6^ cells/mL was used for a further experiment.

### 2.4. MTT Assay

After 90% confluence, the cells were seeded into 96-well plates (5000 cells/well, 100 *μ*L). Cells were treated with different concentrations of A*β*_1–42_ (500 *μ*M, 250 *μ*M, 50 *μ*M, 25 *μ*M, 5 *μ*M, 2.5 *μ*M, 0.5 *μ*M, 0.25 *μ*M, and 0.05 *μ*M) and Goutengsan extract (1 × 10^−1^ g/mL, 2 × 10^−2^ g/mL, 1 × 10^−2^ g/mL, 2 × 10^−3^ g/mL, 1 × 10^−3^ g/mL, 2 × 10^−4^ g/mL, 1 × 10^−4^ g/mL, 2 × 10^−5^ g/mL, and 1 × 10^−5^ g/mL). MTT stock solution of 5.0 mg/mL (100 *μ*L) was added to each well for 4 h to form water-insoluble purple crystal formazan. After removing medium, 100 *μ*L DMSO was added for 10 min microvibration. The absorbance was measured at 570 nm on a microplate reader (Thermo, New York, USA).

### 2.5. Drug Treatment

Cells were treated with basal DMEM medium for control blank group. A*β*_1–42_ treated cells were divided into 5 groups (*n* = 6/group): model group (50 *μ*M A*β*_1–42_); positive control groups (50 *μ*M A*β*_1–42_ and Nimodipine 5 × 10^−6^ mol/L); high dose of Goutengsan group (50 *μ*M A*β*_1–42_ and 2 × 10^−4^ g/mL), medium dose of Goutengsan group (50 *μ*M A*β*_1–42_ and 1 × 10^−4^ g/mL), and low dose of Goutengsan group (50 *μ*M A*β*_1–42_ and 2 × 10^−5^ g/mL). Cells were cultured in an incubator at 37°C with 5% CO_2_ for 24 h.

### 2.6. Apoptosis Assay by Annexin V-Fluorescein Isothiocyanate (FITc)/Propidium Iodide (PI)

PC12 cells were plated on 6-well plates at densities of 5 × 10^5^ cells/well and incubated for 24 h. Basal DMEM medium containing A*β*_1–42_ (50 *μ*M) was added to the wells and incubated for another 24 h prior to the addition of Nimodipine (5 × 10^−6^ mol/L) and Goutengsan extract (2 × 10^−4^ g/mL, 1 × 10^−4^ g/mL, and 2 × 10^−5^ g/mL). Thereafter, these cells were trypsinized, washed with phosphate buffer saline (PBS, pH = 7.4), resuspended in binding buffer, and incubated with staining solution (annexin V/PI = 1 : 2) in the dark for 20 min at room temperature. Immediately, after the annexin V/PI staining, fluorescence-activated cell sorting (FACS) analysis was performed using a FACSCalibur™ flow cytometer (BD Biosciences, San Jose, CA, USA).

### 2.7. Western Blot Analysis

After treating with drug, A*β*_1–42_-induced PC12 cells were washed with PBS. Cell lysates were obtained using ice-cold lysis buffer for proteins extract and were centrifuged with 13,000 ×g for 10 min. Proteins were separated by 10% sodium dodecyl sulfate-polyacrylamide gel (SDS-PAGE) and then transferred onto a Polyvinylidene Fluoride (PVDF) membrane blocking with 5% skim milk. Sequentially, membranes were incubated with the primary antibodies Bax (1 : 200), Bcl-2 (1 : 200), caspase-3 (1 : 200), and caspase-9 (1 : 200) at 4°C. Additionally, membranes were rinsed with Tris Buffered Saline Tween (TBST) for three times (10 min/time) and then incubated with the horseradish peroxidase-bound secondary antibody (1 : 40) in a shaker for 1 h at room temperature. Chemiluminescence reagents were added for the visualization of the protein bands. The quantification of proteins was analyzed by Image-Pro Plus 6.0 software (Media Cybernetics, Inc., Rockville, MD, USA).

### 2.8. Quantitative Real-Time PCR

Total RNA of PC12 cells were extracted by TRIzol reagent. Then it was reverse transcribed with a SuperScript III First-Strand Synthesis System for quantitative real-time polymerase chain reaction (q-PCR) following manufacturer's indications. The sense primer for Bax was 5′-GAGCTGGTGGTTGACTTTCTC-3′ and the antisense primer was 5′-TCCATCTCCGATTCAGTCCCT-3′. The sense primer for caspase-3 was 5′-TAAATGAATGGGCTGAGCTG-3′ and the antisense primer was 5′-ATGGAGAAATGGGCTGTAGG-3′. The sense primer for Bcl-2 was 5′-AATATCCAATCCTGTGCTGCTA-3′ and the antisense primer was 5′-GTCCACGTTCTTCATTGTTACTTC-3′. The sense primer for caspase-9 was 5′- AGGGAAGAGGAGGAGATGAGA-3′ and the antisense primer was 5′-GCTGGGTTTGTCGGTGTT-3′. q-PCR was performed with an ABI 7900 sequence detector (Life Technologies, Carlsbad, CA, USA) using the SYBR Green method and *d*(*N*) 6 random hexamer with primers purchased from Invitrogen. PCR thermocycling parameters were 95°C for 10 min, 40 cycles of 95°C for 15 s, and 60°C for 1 min. Each sample was run in triplicate and was normalized to 18S RNA. Fold changes were determined using the DDCt method. Primer sequences are available upon request.

### 2.9. Animal Model

Female Sprague Dawley (SD) rats (180 ± 20 g) were purchased from SLAC experimental animals Co., Ltd. (Shanghai, China). The animal experiment protocol was reviewed and approved by the Institutional Animal Care and Use Committee of Jiangsu Provincial Academy of Chinese Medicine. Before experimentation, the rats were allowed one-week acclimation period using the independent isolated feeding cages kept at a temperature of 25°C and a relative humidity at 45%. The rats were fed with normal diet and distilled water ad libitum. 20 rats with lateral cerebral ventricle injected with 5 *μ*L A*β*_1–42_ (20 *μ*M) were used for modeling.

### 2.10. Microdialysis Experiment

After modeling for 24 h, the rats were anesthetized with chloral hydrate (300 mg/kg, i.p.) and rat body temperature was maintained at 37°C during the experiment. The blood microdialysis system is composed of the probes for blood CMA/12 microdialysis probe (membrane length, 2 mm; molecular weight cut-off 20 kDa), CMA/402 microinjection pump, and CMA/470 automatic collector (CMA, Stockholm, Sweden). The blood microdialysis probes were fixed in the jugular vein/right atrium and then Ringer's solution (122 mM sodium chloride, 3 mM potassium chloride, 0.4 mM monopotassium phosphate, 1.2 mM magnesium sulfate, 25 mM sodium bicarbonate, and 1.2 mM calcium chloride; pH = 7.0) was perfused at a flow rate of 1 *μ*L/min with the CMA microinjection pump for the postsurgical balancing for 1 h. Then they were orally gavaged with Goutengsan extraction (20 g crude drugs/kg).

Two hours later, the blood microdialysis samples (30 *μ*L) were collected for 15 min, 30 min, 45 min, and 60 min. The samples were added with four times volume of methanol and vortexed for 5 min. To remove the precipitation of proteins, all samples were centrifuged at 11,000 rpm for 10 min. Then the supernatants were evaporated until dryness at room temperature by nitrogen. The residue was dissolved in 100 *μ*L methanol followed by centrifugation at 11,000 rpm for 10 min. At last the samples were analyzed by using the validated UPLC/Q-TOF-MS system.

### 2.11. Ultra-Performance Liquid Chromatography (UPLC)

The UPLC/Q-TOF-MS analysis was performed on a Q-TOF Synapt system (Waters, Milford, MA, USA), equipped with electrospray ionization (ESI) source. Positive ion detection mode was conducted in this analysis. Nitrogen (N_2_), the desolvation gas, was set at the temperature of 350°C with a flow rate of 700 L/h, and the source temperature was 120°C. The capillary and cone voltages were 2.5 kV and 40 V, respectively. The mass data were recorded within the range of 50–1200 Da with a scan time of 0.2 s. The transfer collision energy (*E*_*C*_) was 4 V.

The samples were separated on an Acquity UPLC BEH C_18_ column (50 mm × 2.1 mm, 1.7 *μ*m; Waters, Milford, MA, USA). The mobile phase consisted of acetonitrile (A) and 0.1% formic acid water solution (B). Separation was performed at a flow rate of 1.0 mL/min with gradient elution: from 0 to 2 min, 0–2% A; from 2 to 2.1 min, 2%–6% A; from 2.1 to 4.0 min, 6%–8% A; from 4.0 to 13.0 min, 8%–20% A; 13.0–13.1 min, 20%–2% A; 13.1–14.0 min, 2%-2% A. The injected volume was 3.0 *μ*L. The temperature of autosampler was set at 30°C throughout the analyses. The data was acquired and screened by MassLynx to the next step of the analysis (version 4.1; Waters).

### 2.12. Statistical Analysis

All data were taken from three independent experiments and then expressed as means ± standard deviation (SD). One-way ANOVA was performed to compare the statistical analysis by GraphPad Prism 5.0 (San Diego, CA, USA). Tukey's test was then followed to determine the difference between groups. Statistical significance was indicated by the *p* value which was less than 0.05.

## 3. Results

### 3.1. Cell Viability of Goutengsan on A*β*_*1–42*_-Induced PC12 Cells Injury

**Figure 1:**
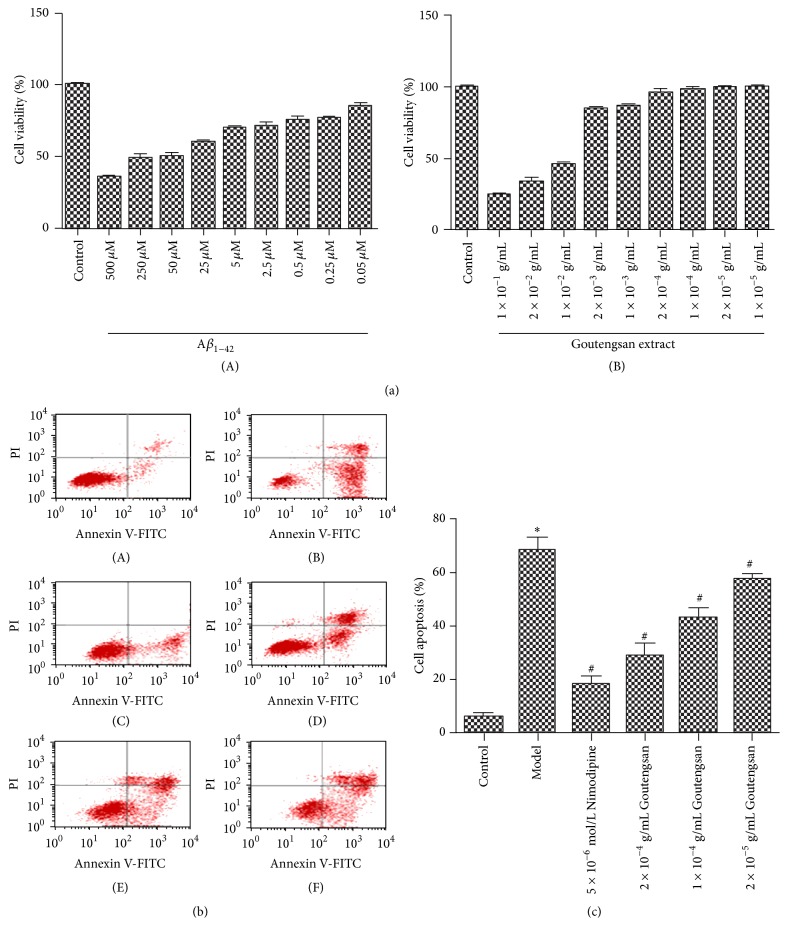
(a) Cell viability of PC12 cells treated with different concentrations of A*β*_1–42_ (A) and Goutengsan extract (B). (b) The effect of Goutengsan on the apoptosis in A*β*_1–42_-induced PC12 cells by annexin V-FITc/PI double-staining analysis. (A) Control group; (B) model group; (C) positive control group (5 × 10^−6 ^mol/L Nimodipine); (D) high dose of Goutengsan group (2 × 10^−4 ^g/mL); (E) medium dose of Goutengsan group (1 × 10^−4 ^g/mL); (F) low dose of Goutengsan group (2 × 10^−5 ^g/mL). (c) Quantitative data of A*β*_1–42_-induced PC12 cells exposed to Goutengsan for 24 hours. The data were shown as mean ± SD, *n* = 6. ^*∗*^*p* < 0.05, control group versus model group. ^#^*p* < 0.05, Nimodipine group versus model group, and Goutengsan group versus model group.

MTT assay was used to determine cell viability for screening the optimal drug concentration of A*β*_1–42_ and Goutengsan extract on PC12 cells. As shown in Figure 1(a), the cell viability ranged from (35.52 ± 2.06)% to (84.74 ± 1.73)% after treatment with different concentration of A*β*_1–42_ (500 *μ*M, 250 *μ*M, 50 *μ*M, 25 *μ*M, 5 *μ*M, 2.5 *μ*M, 0.5 *μ*M, 0.25 *μ*M, and 0.05 *μ*M) while the cell viability was from 24.35 ± 1.45% to 100.30 ± 1.82% after treating with Goutengsan extract (1 × 10^−1^ g/mL, 2 × 10^−2^ g/mL, 1 × 10^−2^ g/mL, 2 × 10^−3^ g/mL, 1 × 10^−3^ g/mL, 2 × 10^−4^ g/mL, 1 × 10^−4^ g/mL, 2 × 10^−5^ g/mL, and 1 × 10^−5^ g/mL). Therefore, we chose A*β*_1–42_ in 50 *μ*M and Goutengsan extract in 2 × 10^−4^ g/mL, 1 × 10^−4^ g/mL, and 2 × 10^−5^ g/mL for the further study. Furthermore, Goutengsan has a protective effect on A*β*_1–42_-induced PC12 cells injury.

### 3.2. Antiapoptosis of Goutengsan in A*β*_*1–42*_-Induced PC12 Cells

Cell apoptosis was assessed by flow cytometry using the annexin V-FITC/PI assay kit in accordance with the manufacture's protocols. As shown in Figures 1(b) and 1(c) comparing with control blank group, the percentage of apoptotic cells was significantly increased in model group (*F* = 2.88 × 10^7^, *df* = 1, *p* = 3.47 × 10^−8^ < 0.05). Nevertheless, after treating with Nimodipine (5 × 10^−6^ mol/L), the percentage of apoptotic cells was significantly decreased (*F* = 8.04 × 10^6^, *df* = 1, *p* = 1.24 × 10^−7^ < 0.05). A*β*_1–42_-induced PC12 cells treated with Goutengsan extract (2 × 10^−4 ^g/mL, 1 × 10^−4 ^g/mL, and 2 × 10^−5 ^g/mL) compared with model group were also significantly decreased (*F* = 6781.39, *df* = 3, *p* = 5.61 × 10^−11^ < 0.05) in a concentration-dependent manner, which indicated that Goutengsan had a protective effect on A*β*_1–42_-induced PC12 cell apoptosis.

**Figure 2:**
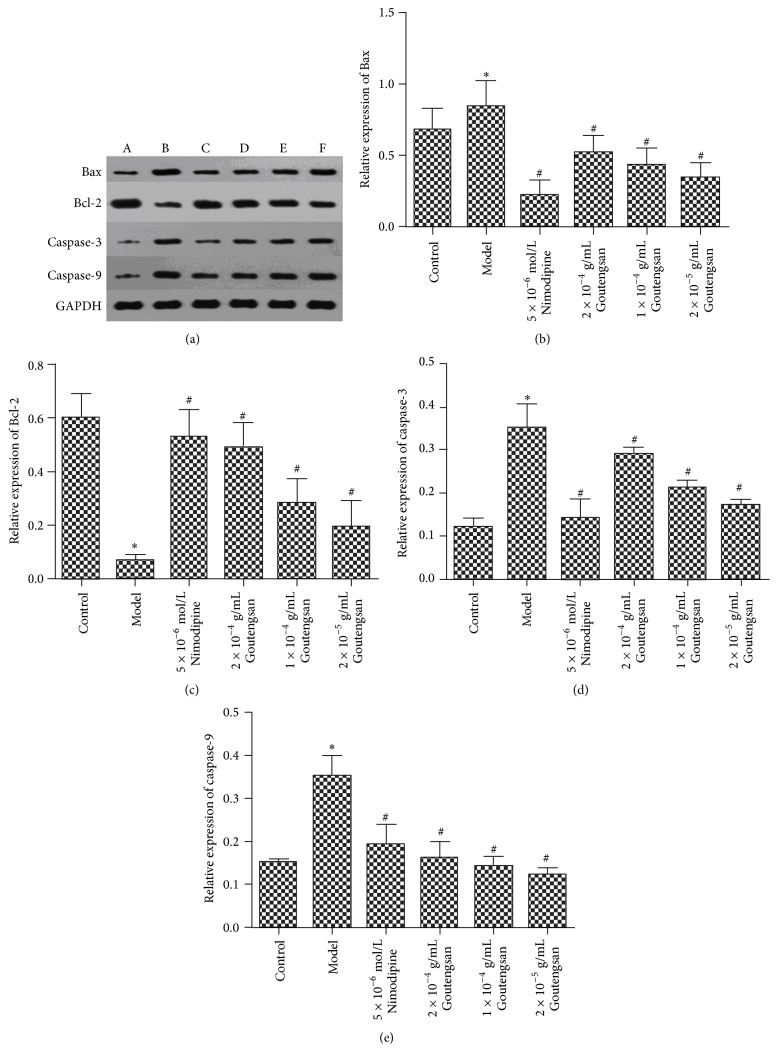
Western blot analysis for protein expression in A*β*_1–42_-induced PC12 cells. (a) A*β*_1–42_-induced PC12 cells were treated with Goutengsan and protein levels were analyzed by western blotting. A: control group; B: model group; C: positive control group (5 × 10^−6 ^mol/L Nimodipine); D: high dose of Goutengsan group (2 × 10^−4 ^g/mL); E: medium dose of Goutengsan group (1 × 10^−4 ^g/mL); F: low dose of Goutengsan group (2 × 10^−5 ^g/mL). (b) Bax protein level; (c) Bcl-2 protein level; (d) caspase-3 protein level; (e) caspase-9 protein level. The data were shown as mean ± SD, *n* = 3. ^*∗*^*p* < 0.05, control group versus model group. ^#^*p* < 0.05, Nimodipine group versus model group, and Goutengsan group versus model group.

For further assessment of cell apoptosis in Goutengsan, we performed western blotting on protein expression analysis. As shown in Figure 2, comparing with the control blank group, the expression of Bcl-2 was significantly decreased (*p* < 0.05, Table 2), while the expression of Bax, caspase-3, and caspase-9 was remarkably increased in model group (*p* < 0.05, Table 2). However, after treating with Nimodipine (5 × 10^−6^ mol/L) and Goutengsan extract (2 × 10^−4^ g/mL, 1 × 10^−4^ g/mL, and 2 × 10^−5^ g/mL), the overexpression of Bax, caspase-3, and caspase-9 was significantly reduced (*p* < 0.05, Table 2) and the expression of Bcl-2 was obviously enhanced (*p* < 0.05, Table 2). The results demonstrated that Goutengsan could regulate A*β*_1–42_-induced PC12 cells apoptosis via inhibiting the expression of Bax, caspase-3, and caspase-9, but enhancing the expression of Bcl-2.

**Figure 3:**
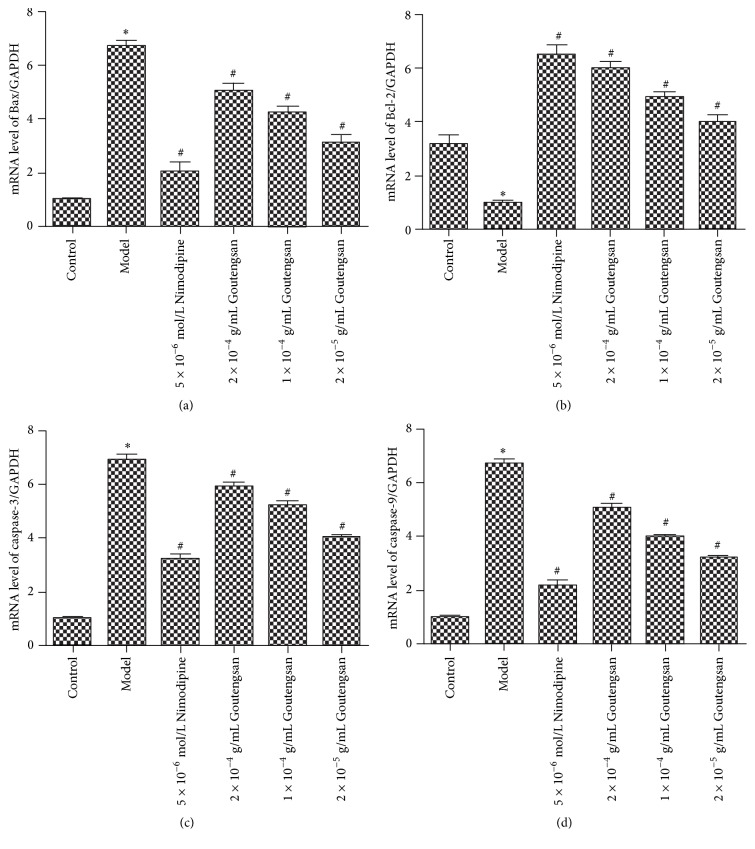
Effects of Goutengsan on the mRNA levels of Bax (a), Bcl-2 (b), caspase-3 (c), and caspase-9 (d) in A*β*_1–42_-induced PC12 cells. All experiments were repeated at least three times. Data were represented as mean ± SD (*n* = 6). ^*∗*^*p* < 0.05, control group versus model group. ^#^*p* < 0.05, Nimodipine group versus model group, and Goutengsan group (2 × 10^−4 ^g/mL, 1 × 10^−4 ^g/mL, 2 × 10^−5 ^g/mL) versus model group.

To further explain the results above, q-PCR was also used to determine the mRNA levels of Bax, Bcl-2, caspase-3, and caspase-9 for evaluating cell apoptosis. As described in Figure 3, comparing to control blank group, the mRNA levels of Bax, caspase-3, and caspase-9 in model group were significantly increased (*p* < 0.05, Table 2), while Bcl-2 mRNA level was significantly decreased (*p* < 0.05, Table 2). However, Nimodipine (5 × 10^−6 ^mol/L) and Goutengsan extract (2 × 10^−4^ g/mL, 1 × 10^−4^ g/mL, and 2 × 10^−5^ g/mL) significantly decreased the mRNA levels of Bax, caspase-3, and caspase-9 (*p* < 0.05, Table 2) comparing with model group, but the mRNA level of Bcl-2 was significantly increased (*p* < 0.05, Table 2). The results also indicated that Goutengsan could regulate A*β*_1–42_-induced PC12 cells apoptosis via reducing the mRNA levels of Bax, caspase-3, and caspase-9, but increasing the mRNA level of Bcl-2.

### 3.3. Identification of Components in Rat Blood Samples

**Figure 4:**
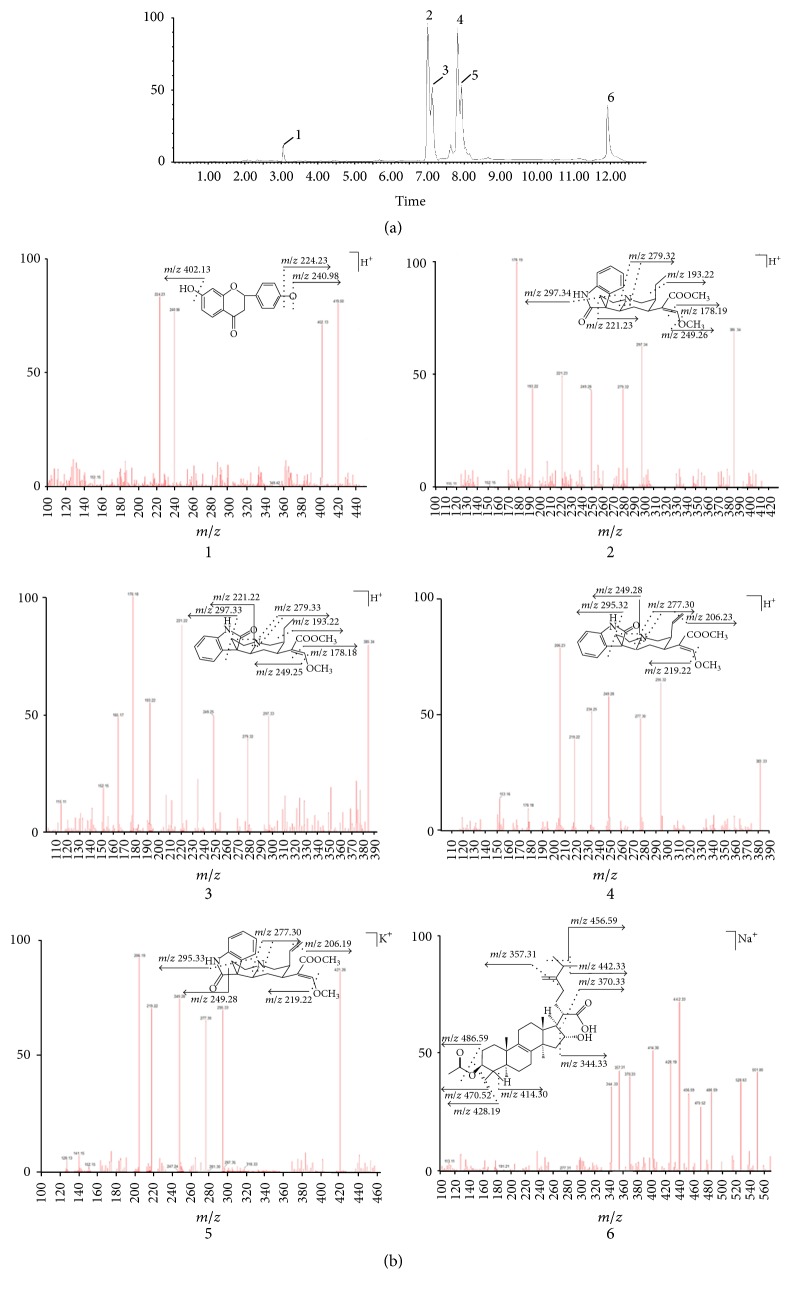
(a) UPLC chromatogram of Goutengsan in rat blood samples. (b) Mass spectra of 6 metabolites in rat blood samples. 1, liquiritin (Chemical Structure: C_21_H_22_O_9_, MW: 418.39); 2, rhynchophylline (chemical structure: C_21_H_22_O_9_, MW: 384.47); 3, isorhynchophylline (Chemical Structure: C_21_H_22_O_9_, MW: 384.47); 4, corynoxeine (chemical structure: C_21_H_22_O_9_, MW: 382.45); 5, isocorynoxeine (Chemical Structure: C_21_H_22_O_9_, MW: 382.45); 6, pachymic acid (chemical structure: C_21_H_22_O_9_, MW: 418.39).

After being sampled with MD, the main compounds in rat plasma were analyzed and identified by Ultra-Performance Liquid Chromatography (UPLC). UPLC chromatogram of Goutengsan was shown in Figure 4(a). The chromatogram showed that the sample could be eluted completely under the used UPLC conditions within 14 min and separated satisfactorily. Six components (1–6) (Figure 4(b)) in plasma were identified and tentatively characterized by UPLC/Q-TOF-MS. The results indicated that six components (1–6) could be absorbed into blood.

Liquiritin (1, 11.23 *μ*g/mL) whose molecular formula was determined to be C_21_H_22_O_9_ displayed a series of quasi-molecular ion peak at* m/z* 402.13 [M + H^+^  − OH]^−^,* m/z* 240.98 [M + H^+^  − glc]^−^, and* m/z* 224.23 [M + H^+^  − O]^−^.

Rhynchophylline (2, 1.46 *μ*g/mL), C_21_H_22_O_9_, displayed a series of quasi-molecular ion peak at* m/z* 297.33 [M + H^+^  − C_6_H_7_N]^−^,* m/z* 279.33 [M + H^+^  − NH_3_]^−^,* m/z* 249.25 [M + H^+^  − CH_3_O]^−^,* m/z* 221.22 [M + H^+^  − O]^−^,* m/z* 193.22 [M + H^+^  − C_2_H_5_]^−^, and* m/z* 178.18 [M + H^+^  − C_2_H_3_]^−^.

Isorhynchophylline (3, 10.67 *μ*g/mL), C_21_H_22_O_9_, displayed a series of quasi-molecular ion peak at* m/z* 297.34 [M + H^+^  − C_6_H_7_N]^−^,* m/z* 279.32 [M + H^+^  − NH_3_]^−^,* m/z* 249.26 [M + H^+^  − CH_3_O]^−^,* m/z* 221.33 [M + H^+^  − O]^−^,* m/z* 193.22 [M + H^+^  − C_2_H_5_]^−^, and* m/z* 178.19 [M + H^+^  − C_2_H_3_]^−^.

Corynoxeine (4, 1.35 *μ*g/mL), C_21_H_22_O_9_, displayed a series of quasi-molecular ion peak at* m/z* 295.32 [M + H^+^  − C_6_H_7_N]^−^,* m/z* 277.30 [M + H^+^  − NH_3_]^−^,* m/z* 249.28 [M + H^+^  − O]^−^, and* m/z* 219.22 [M + H^+^  − CH_3_O]^−^.

Isocorynoxeine (5, 12.83 *μ*g/mL), C_21_H_22_O_9_, displayed a series of quasi-molecular ion peak at* m/z* 295.33 [M + H^+^  − C_6_H_7_N]^−^,* m/z* 277.32 [M + H^+^  − NH_3_]^−^,* m/z* 249.28 [M + H^+^  − O]^−^,* m/z* 219. 22 [M + H^+^  − CH_3_O]^−^, and* m/z* 206.19 [M + H^+^  − CH_2_]^−^.

Pachymic acid (6, 9.83 *μ*g/mL), C_21_H_22_O_9_, displayed a series of quasi-molecular ion peak at* m/z* 486.59 [M + Na^+^  − C_2_H_3_O]^−^,* m/z* 470.52 [M + Na^+^  − O]^−^,* m/z* 456.59 [M + Na^+^  − CH_3_]^−^,* m/z* 442.33 [M + Na^+^  − CH_3_]^−^,* m/z* 428.19 [M + Na^+^  − CH_3_]^−^,* m/z* 414.30 [M + Na^+^  − CH_3_]^−^,* m/z* 370.33 [M + Na^+^  − CHO_2_]^−^, and* m/z *357.31 [M + Na^+^  − CH_2_]^−^.

### 3.4. Protection of Six Components on A*β*_*1–42*_-Induced PC12 Cells

**Figure 5:**
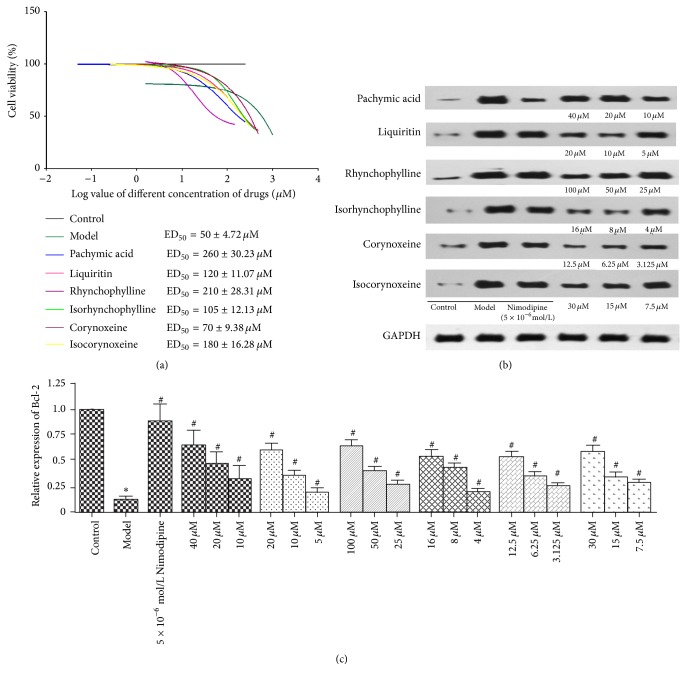
(a) ED_50_ curved line of pachymic acid, liquiritin, rhynchophylline, isorhynchophylline, corynoxeine, and isocorynoxeine in PC12 cells. (b) A*β*_1–42_-induced PC12 cells were treated with six components (pachymic acid, liquiritin, rhynchophylline, isorhynchophylline, corynoxeine, and isocorynoxeine) and protein levels were analyzed by western blotting. (c) Bcl-2 protein level. The data were shown as mean ± SD, *n* = 3. ^*∗*^*p* < 0.05, control group versus model group. ^#^*p* < 0.05, Nimodipine group versus model group, and six components' group versus model group.

To verify the role of components (pachymic acid, liquiritin, rhynchophylline, isorhynchophylline, corynoxeine, and isocorynoxeine) identified in rat plasma on cell apoptosis, we used western blotting to evaluate the protein expression. As can be seen from Figures 5(b) and 5(c), comparing with the control blank group, the expression of Bcl-2 in model group was significantly decreased (*F* = 5.34 × 10^4^, *df* = 1, and *p* = 1.87  × 10^−5^ < 0.05). However, after treating with Nimodipine (5 × 10^−6^ mol/L; *F* = 2.03 × 10^4^, *df* = 1, and *p* = 4.92  ×  10^−5^ < 0.05), pachymic acid (40 *μ*M, 20 *μ*M, and 10 *μ*M; *F* = 203.35, *df* = 3, and *p* = 2.01  × 10^−6^ < 0.05), liquiritin (20 *μ*M, 10 *μ*M, and 5 *μ*M; *F* = 120.07, *df* = 3, and *p* = 9.56  × 10^−6^ < 0.05), rhynchophylline (25 *μ*M, 50 *μ*M, and 100 *μ*M; *F* = 7.49  × 10^3^, *df* = 3, and *p* = 4.16  ×  10^−11^ < 0.05), isorhynchophylline (16 *μ*M, 8 *μ*M, and 4 *μ*M; *F* = 3.64 × 10^3^, and *df* = 3, *p* = 3.63  × 10^−10^ < 0.05), corynoxeine (12.5 *μ*M, 6.25 *μ*M, and 3.125 *μ*M; *F* = 105.79, and *df* = 3, *p* = 1.39  × 10^−5^ < 0.05), and isocorynoxeine (30 *μ*M, 15 *μ*M, and 7.5 *μ*M; *F* = 780.81, and *df* = 3, *p* = 3.64  × 10^−8^ < 0.05) (Figure 5(a)), the protein expression of Bcl-2 was significantly increased comparing with model group. Thus, it appears that pachymic acid, liquiritin, rhynchophylline, isorhynchophylline, corynoxeine, and isocorynoxeine could regulate A*β*_1–42_-induced PC12 cells apoptosis via enhancing the expression of Bcl-2.

## 4. Discussion

Goutengsan, a widely used Chinese herbal formula, was first recorded in “Effective Prescriptions for Universal Relief” (Chinese name: Benshi Fang) for chronic headache and hypertension. At present, our researches of Goutengsan were mainly focused on the effect of A*β* in brain for AD treatment. As a Chinese herbal formula, Goutengsan consisted of so more medicinal herbs that it is difficult to study its complicated composition. Different analysis techniques should be explored for rapid analysis of Chinese herbal formula.

Microdialysis (MD) is a powerful sampling technique that enables monitoring of dynamic processes* in vitro* and* in vivo* [16]. MD can be used for researching the diseases, pathology, and therapeutic methods in the fields of neurotransmitters coupled with all kinds of analysis technique for quantitative analysis [17]. The applications of MD and its hyphenated techniques play an important role in the analysis of endogenous substances, pharmacokinetic study of Traditional Chinese Medicine (TCM), and drug interactions [18]. MD combined with chromatographic or electrophoretic methods had an extensive application in recent years. Therefore, in order to analyze the components of Goutengsan in rat plasma, MD coupling with UPLC/Q-TOF-MS was used for sampling. We inferred that Goutengsan at least pachymic acid, liquiritin, rhynchophylline, isorhynchophylline, corynoxeine, and isocorynoxeine could be absorbed into blood.

Amyloid-beta (A*β*) peptides, especially A*β*_1–42_, cause neurotoxicity and cell death in the brain of Alzheimer's disease (AD) at higher concentrations [19]. Some soluble A*β* oligomers, highly prone to aggregation and self-assemble to form a heterogeneous mixture of oligomers and protofibrils, are considered as the major neurotoxic species in AD [20–22]. A*β* induced apoptosis occurs in the AD brain and plays a role in neuronal dysfunction and neurodegeneration, which contributes to the progression of AD [23]. In this study, A*β*_1–42_ exposed PC12 cells were used to investigate the protection mechanism of Goutengsan* in vitro* and rats lateral cerebral ventricle injected with A*β*_1–42_ was used* in vivo*.

Executioner caspases (caspase-3, caspase-6, and caspase-7) and upstream effector caspases (caspase-2, caspase-8, caspase-9, and caspase-10) are well known as mediators in AD brains [23]. Caspase-3 and caspase-9 may also be potential therapeutic targets for AD [17]. It is well known that caspase-9 can activate caspase-3. Activated caspase-3 cleaves poly ADP-ribose polymerase (PARP) that helps cells to maintain their viability. Caspase-3 is also demonstrated to cleave amyloid precursor protein (APP) and tau [24]. Caspase-9 is reported to mediate a fall in synaptic plasticity and memory deficits [25]. A change of Bax/Bcl-2 ratio and subsequent activation of caspase-9 play a central role in the apoptosome-dependent intrinsic pathway of apoptosis [26]. Western blotting and q-PCR were used to evaluate the cell apoptosis in this experiment. Our results showed that Goutengsan could upregulate the expression of Bcl-2 and downregulate the expression of Bax, caspase-9, and caspase-3. In addition, Goutengsan could also regulate A*β*_1–42_-induced PC12 cells apoptosis via reducing Bax, caspase-9, and caspase-3 levels, while increasing Bcl-2 level.

As a Chinese herbal formula, Goutengsan consisted of 11 medicinal herbs. In the present study, 6 components from 3 medicinal herbs of Goutengsan were identified in rat plasma by MD sampling. This does not mean that other components may not play a role in the protective effect on A*β*_1–42_-induced cell death. We just found these components because of the limitation of analytical approach for finding low content components existing in MD samples. And some components may change into other components or improve the absorption of other components. Four components including rhynchophylline, isorhynchophylline, corynoxeine, and isocorynoxeine were main alkaloids in Gouteng, while pachymic acid and liquiritin were the components from Fuling and Gancao, respectively. Early exploratory research indicated that alkaloids of Goutengsan had a protective effect on AD [9, 10]. So we inferred that six components (pachymic acid, liquiritin, rhynchophylline, isorhynchophylline, corynoxeine, and isocorynoxeine) identified in rat plasma were effective components in Goutengsan. At last, western blotting was used for evaluating the protection of six components identified in rat plasma on A*β*_1–42_ induced PC12 cells; the result suggested that six components could protect A*β*_1–42_-induced PC12 cells from apoptosis via upregulating the expression of Bcl-2 and downregulating the expression of caspase-3.

## 5. Conclusion

In conclusion, Goutengsan has a protective effect on A*β*_1–42_-induced PC12 cells as well as 6 components (pachymic acid, liquiritin, rhynchophylline, isorhynchophylline, corynoxeine, and isocorynoxeine) which could be absorbed into blood via reducing cell damage and increasing the vitality of the cells. Thus, the results turned out that pachymic acid, liquiritin, rhynchophylline, isorhynchophylline, corynoxeine, and isocorynoxeine were effective components for the treatment of AD. This study provides pharmacological application of Goutengsan for the treatment of AD.

## Supplementary Material

The Supplementary Material was the determination of major components in MD samples including preparation of stock solutions, preparation of standard solutions and validation procedure (linearity, intra-day precision, inter-day precision and recovery).

## Figures and Tables

**Table 1:** Description of Goutengsan.

Chinese name	Latin name	Plant part	Processing	Genus	Batch number
Gou teng	*Uncaria rhynchophylla (Miq.) Miq. ex Havil.*	Stem	Dried	*Uncaria*	20140415
Chenpi	*Citrus reticulata Blanco*	Pericarp	Dried	*Citrus*	20150502
Maidong	*Ophiopogon japonicus*	Earthnut	Dried	*Ophiopogon*	20140116
Banxia	*Pinellia ternata (Thunb.) Breit.*	Earthnut	Lime and liquorice	*Pinellia*	20150321
Fuling	*Poria cocos (Schw.) Wolf*	Sclerotium	Dried	*Wolfiporia*	20130705
Renshen	*Panax ginseng C. A. Mey.*	Earthnut	Dried	*Panax*	20150317
Fangfeng	*Saposhnikovia divaricata (Turcz.) Schischk*	Rhizome	Dried	*Saposhnikovia*	20150219
Juhua	*Dendranthema morifolium (Ramat.) Tzvel.*	Inflorescence	Dried	*Chrysanthemum*	20150526
Shengjiang	*Zingiber officinale Roscoe*	Earthnut	Dried	*Zingiber*	20140211
Gancao	*Glycyrrhiza uralensis Fisch.*	Rhizome	Dried	*Glycyrrhiza*	20150321
Shigao	*Gypsum*	—	Grinded	—	20150219

**Table 2:** Mathematical statistics of western blot and PCR.

	Group	Bcl-2			Bax			Caspase-3			Caspase-9		
		*F*	*df*	*p*	*F*	*df*	*p*	*F*	*df*	*p*	*F*	*df*	*p*
Western blot	Model group versus control group	3.13 × 10^3^	1	3.19 × 10^−4^	36.60	1	2.60 × 10^−2^	1.06 × 10^4^	1	9.38 × 10^−5^	9.2 × 10^4^	1	1.01 × 10^−5^
	Nimodipine group versus model group	1.45 × 10^3^	1	1.00 × 10^−3^	315.00	1	3.00 × 10^−3^	5.35 × 10^3^	1	1.87 × 10^−4^	738.00	1	1.00 × 10^−3^
	Goutengsan group versus model group	309.00	3	5.78 × 10^−7^	533.00	3	1.14 × 10^−7^	440.00	3	2.02 × 10^−7^	1.35 × 10^3^	3	7.14 × 10^−9^

PCR	Model group versus control group	7.03 × 10^3^	1	1.42 × 10^−4^	8.28 × 10^3^	1	1.21 × 10^−4^	4.33 × 10^4^	1	2.31 × 10^−5^	1.82 × 10^4^	1	5.5 × 10^−5^
	Nimodipine group versus model group	9.19 × 10^3^	1	1.09 × 10^−4^	7.42 × 10^3^	1	1.35 × 10^−4^	1.85 × 10^4^	1	5.41 × 10^−5^	1.07 × 10^4^	1	9.35 × 10^−5^
	Goutengsan group versus model group	4.16 × 10^3^	3	2.43 × 10^−10^	2.79 × 10^3^	3	8.07 × 10^−10^	5.69 × 10^3^	3	9.5 × 10^−11^	1.96 × 10^3^	3	2.3 × 10^−9^
