# Supplementary material for: Components of Goutengsan in Rat Plasma by Microdialysis Sampling and Its Protection on Aβ1–42-Induced PC12 Cells Injury
Source: Evid Based Complement Alternat Med. 2017 Mar 2;2017:7593027. doi: 10.1155/2017/7593027 (PMC5352969; doi:10.1155/2017/7593027)
Supplement: Supplementary file 1 — The Supplementary Material was the determination of major components in MD samples including preparation of stock solutions, preparation of standard solutions and validation procedure (linearity, intra-day precision, inter-day precision and recovery). [file 7593027.f1.docx]

**Determination of major components in MD samples**

**Preparation of Stock Solutions**

The samples were added with four times volume of methanol and vortexed for 5 min. To remove the precipitation of proteins, all samples were centrifuged at 11,000 rpm for 10 min. Then the supernatants were evaporated until dryness at room temperature by nitrogen. The residue was dissolved in 100 μL methanol followed by centrifugation at 11,000 rpm for 10 min.

**Preparation of Standard Solutions**

A stock solution of pachymic acid, liquiritin, rhynchophylline, isorhynchophylline, corynoxeine and isocorynoxeine was prepared in methanol and kept below 4 ℃. Standard solutions were prepared by serial dilution of the stock solution to 30 μg/mL.

**Validation Procedure**

Linearity was tested using six different amounts of compounds pachymic acid (I), liquiritin (II), rhynchophylline (III), isorhynchophylline (IV), corynoxeine (V), isocorynoxeine (VI), in the ranges 5.73-91.17, 2.41-106.81, 2.78-85.02, 3.05-238.01，1.09-30.60, 14.67-292.76 μg/mL, respectively. Solutions corresponding to each concentration level were injected in triplicate and linear regression analysis of the ingredients. Peak area (Y) versus the concentration (X) was carried out to obtain linear regression equation. Correlation coefficient (R^2^ > 0.9990) of calibration curves obtained from each analyte indicated that there was a strong linear relationship between peak area and concentrations (Table 1).

Intra-day precision of the developed method was evaluated by repeating six determinations of sample solution on the same day. Inter-day precision was also determined by analyzing the standard solution on three consecutive days as well. The results indicated that the instrument and the method had a good precision (Table 2). Additionally, six samples were analyzed to determine the repeatability of the proposed method. It was showed good repeatability of this method due to the RSD of peak areas of all ingredients were less than 1.59 % (Table 2). Sample solution was injected at room temperature for 0, 2, 4, 8, 12 and 24 h. The results given in Table 2 revealed that the sample solution was found to be stable within 24 h.

A recovery test was conducted to evaluate the accuracy of the developed method. The standards dissolved in methanol were added into the six samples which the contents were known. Then methanol was added to proper volume of 10 mL. The sample solutions at each kind were in triplicate and the recovery percentage was calculated based on the following formula:

Recovery = (Detected amount – Original amount) / (Spiked amount) × 100 %

The results were presented in Table 3 and the recoveries of the ingredients were within the range of 95.8-105.0 % and RSD values ranging from 1.22 % to 2.09 %, verifying the high accuracy of the method.

**Table 1** Calibration curves of the six ingredients

| **NO.** | **Ingredients** | **Range (****μg/mL)** | **Equation for regression line** | **R^2^** |
| --- | --- | --- | --- | --- |
| I | pachymic acid | 5.73～91.17 | Y=191X-0.95 | 0.9990 |
| II | liquiritin | 2.41～106.81 | Y=189X+0.05 | 0.9998 |
| III | rhynchophylline | 2.78～85.02 | Y=171X-11.51 | 0.9995 |
| IV | isorhynchophylline | 3.05～238.01 | Y=884X+0.63 | 0.9999 |
| V | corynoxeine | 1.09～30.60 | Y=180X-5.59 | 0.9998 |
| VI | isocorynoxeine | 14.67～292.76 | Y=187X+0.08 | 0.9998 |

**Table 2** Precision Tests and Stability of Sample Solution (RSD (%))

| **NO.** | | **Ingredients** | | **Intra-Day Precision (n=6)** | **Inter-Day Precision (n=3)** | **Repeatability**  **(n=6)** | **Stability**  **(n=6)** |
| --- | --- | --- | --- | --- | --- | --- | --- |
| I | | pachymic acid | | 0.38 | 1.22 | 1.46 | 0.46 |
| II | | liquiritin | | 1.03 | 1.31 | 1.21 | 0.86 |
| III | | rhynchophylline | | 0.52 | 0.61 | 1.33 | 1.23 |
| IV | isorhynchophylline | | 1.20 | | 0.42 | 1.09 | 1.45 |
| V | corynoxeine | | 1.33 | | 0.98 | 1.18 | 1.11 |
| VI | isocorynoxeine | | 0.53 | | 1.07 | 1.32 | 0.83 |

**Table 3** Content and Recoveries of the six ingredients (n=3)

| **NO.** | **Ingredients** | **Content (μg/mL)** | **Recovery Mean (%)** | **RSD (%)** |
| --- | --- | --- | --- | --- |
| I | pachymic acid | 9.83 | 99.00 | 1.29 |
| II | liquiritin | 11.23 | 98.50 | 1.22 |
| III | rhynchophylline | 1.46 | 97.80 | 1.81 |
| IV | isorhynchophylline | 10.67 | 101.20 | 1.67 |
| V | corynoxeine | 1.35 | 96.46 | 2.09 |
| VI | isocorynoxeine | 12.83 | 96.78 | 1.99 |
